# Prediction of Anxiety and Depression Using Preconcussion Screening Assessments in Collegiate Athletes

**DOI:** 10.1155/tsm2/6927094

**Published:** 2026-02-13

**Authors:** Chase R. Siewert, Caroline J. Ketcham, Eric E. Hall

**Affiliations:** ^1^ Department of Exercise Science and Elon BrainCARE Research Institute, Elon University, Elon, North Carolina, USA, elon.edu

**Keywords:** ADHD, collegiate athletes, concussion symptoms, ImPACT, mental health

## Abstract

Accurate identification of anxiety and depression in collegiate athletes is critical to timely intervention. Baseline concussion testing, such as the Immediate Post‐Concussion Assessment and Cognitive Testing (ImPACT), may provide a unique, practical avenue for screening mental health concerns. The objective of this study was to determine whether the symptom cluster scores from ImPACT can predict anxiety and depression in collegiate athletes. 560 athletes (43.7% female; 16% ADHD) completed baseline assessments preseason. Symptom clusters (e.g., affective) were derived from ImPACT and compared with scores on the Generalized Anxiety Disorder‐7 (GAD‐7) and Patient Health Questionnaire‐9 (PHQ‐9). Hierarchical regression analyses examined whether ImPACT clusters and demographic factors could predict anxiety and depression. Gender and ADHD explained a small but significant portion of the variance in depression (*R* ^2^ change = 0.040, *p* < 0.001), cognitive clusters (*R* ^2^ change = 0.018, *p* < 0.001), and affective clusters (*R* ^2^ change = 0.259, *p* < 0.001). In the prediction of anxiety, the affective cluster was again the strongest predictor, contributing to a substantial increase in explained variance (*R* ^2^ change = 0.257, *p* < 0.001), followed by demographic factors, sex, and ADHD, which explained a smaller portion of the variance (*R* ^2^ change = 0.035, *p* < 0.001). ImPACT concussion symptom clusters appear to be a useful tool for preemptive screening of anxiety and depression in collegiate athletes. Including this utility in existing concussion protocols, which ubiquitously use ImPACT, may facilitate earlier identification of at‐risk individuals for a more targeted intervention to support student‐athletes’ psychological well‐being.

## 1. Introduction

Mental health among collegiate athletes has become an increasingly significant area of concern, with anxiety and depression affecting a growing number of students in this population. According to the NCAA’s 2020 well‐being survey, 30% of female athletes and 25% of male athletes report symptoms of anxiety, yet only 10% of student‐athletes seek professional help [1]. These findings parallel broader college student trends, where stress, academic pressures, and social challenges contribute to elevated mental health risks [2]. However, student‐athletes face additional demands—intense training regimens, performance expectations, and a highly competitive environment—that compound these risks. Research suggests that these unique stressors not only increase susceptibility to mental health conditions but also shape how symptoms manifest and are reported [3, 4]. Gouttebarge and colleagues found that implementing a standardized mental health assessment tool, modeled after existing concussion protocols, helped detect anxiety, depression, and other related mental health disorders earlier in Olympic athlete populations, facilitating timely clinical management [5]. Their development of the Sport Mental Health Assessment Tool (SMHAT‐1) demonstrates the power of systematic screening in reducing stigma, elucidating previously hidden symptoms, and expediting access to support services. Applied to a collegiate setting, this approach demonstrates the merit of integrating mental health measures into routine athletic health evaluations.

Despite the growing need for mental health services in collegiate sports, underreporting remains a persistent challenge. The stigma surrounding mental health, particularly in high‐performance cultures, can deter athletes from disclosing symptoms for fear of being perceived as weak or losing playing time [6]. In addition, conventional assessments often rely on self‐reported questionnaires designed for the general population, which may overlook the subtle ways in which athletes normalize fatigue, stress, or physical discomfort [7]. Consequently, standard screening tools may underestimate true levels of psychological distress in this group.

Several studies have highlighted the importance of tailored, sport‐specific screening protocols that capture the complex interplay between physical and psychological stressors [8–10]. One promising avenue involves the repurposing of existing concussion assessment tools, which are already embedded in many collegiate athletic programs [10, 11]. The Immediate Post‐Concussion Assessment and Cognitive Testing (ImPACT) was originally designed to evaluate neurocognitive function after head injuries, but it also can elucidate four major symptom clusters—affective (i.e., sadness, irritability), cognitive (i.e., concentration difficulties), physical (i.e., headaches and dizziness), and sleep (i.e., disruptions in sleep patterns) [10]. Each cluster reflects a set of self‐reported symptoms, all of which are rated by athletes to produce cluster‐specific symptom scores. Riegler and colleagues reported that the ImPACT Post‐Concussion Symptom Scale affective cluster demonstrated convergent validity with standardized depression measures in collegiate athletes [10]. LoGalbo, DaCosta, and Webbe compared Patient Health Questionnaire‐9 (PHQ‐9) scores with ImPACT symptom clusters and found meaningful construct overlap with a validated depression evaluation [11]. Together, these studies suggest that using ImPACT symptom clusters to transform ImPACT from a purely concussion‐focused protocol into an integrated mental health screening tool is psychometrically defensible.

This study aims to evaluate the predictive power of ImPACT symptom clusters in identifying anxiety and depression among collegiate athletes. Beyond simply tracking symptom changes postconcussion, we investigate how the affective, cognitive, physical, and sleep clusters relate to broader mental health indicators. By comparing these cluster‐specific scores with standardized measures of depression and anxiety, we seek to determine whether this widely used assessment can detect early warning signs of psychological distress. In our framework, elevated ImPACT affective or sleep cluster scores serve as screening flags that may prompt administration of more reliable measures: PHQ‐9 for depression and Generalized Anxiety Disorder‐7 (GAD‐7) for anxiety, after which athletes who meet moderate or higher thresholds are referred to care according to institutional protocols.

Furthermore, we incorporate demographic factors—particularly gender and ADHD status—since recent literature has highlighted their role in shaping mental health outcomes among athletes. In particular, Cottle et al. observed that student‐athletes with preexisting factors such as ADHD or migraines often demonstrated poorer performance on neurocognitive tasks and reported higher symptom scores on ImPACT [12]. These findings illustrate the significance of evaluating baseline data and considering comorbidities when evaluating both concussion and mental health concerns. Female athletes often report higher rates of anxiety and depressive symptoms [4], and multiple studies indicate that they experience more intense or prolonged concussion symptoms—such as headaches, dizziness, and emotional lability—further elevating mental health risks [13, 14]. Meanwhile, individuals with ADHD may experience exacerbated stress responses and difficulties in executive function that can mask or intensify mental health issues [15, 16].

These considerations reflect a broader shift within sports medicine toward comprehensive athlete health, recognizing that cognitive, emotional, and physical well‐being are intertwined [17]. Although concussion evaluation protocols are widely implemented, few studies have leveraged their symptom‐tracking capabilities for mental health screening. This study seeks to broaden the scope of existing concussion assessments by positioning ImPACT as a potentially valuable resource for detecting anxiety and depression among collegiate athletes. By examining the relationships between ImPACT’s cluster‐specific symptom scores, demographic variables, and mental health indicators, we propose a scalable approach that aligns with ongoing calls for more integrated, proactive monitoring. Ultimately, integrating ImPACT‐based mental health assessments into routine athletic protocols could help address barriers to care, reduce underreporting, and promote earlier interventions for student‐athletes at risk.

## 2. Methods

### 2.1. Participants

The study involved 560 varsity and club athletes (276 females, 49.3%; mean age = 18.7 ± 1.0; see Table 1 for more information about the demographics of the sample) from a diverse array of sports (see Table 2 for a listing of sports) at a mid‐sized private university in the Southeast portion of the United States. Prior to their athletic seasons, all participants were required to complete baseline assessments in a controlled test environment to support their care by sports medicine staff. Participants were given the opportunity to opt into their baseline data being used for research purposes. All participants included in this study opted in and completed an informed consent form approved by the university’s institutional review board.

**TABLE 1:** Demographics for sample.

Descriptor	Number
*Race*	
American Indian or Alaskan Native	4
Asian	10
Black or African American	52
Hispanic or Latino	15
Native Hawaiian or Pacific Islander	10
White	428
Multiracial or other	34
Missing	7
*International*	
Yes	34
No	518
Missing	8

*Year in school*	
1^st^ year	338
Sophomore	139
Junior	45
Senior	22
Completed bachelor’s degree or graduate program	5

**TABLE 2:** Sport participation of the sample.

Sport	Number
Baseball	38
Basketball	23
Dance	29
Equestrian sports	6
Field hockey	7
Football	39
Golf	7
Ice hockey	8
Lacrosse	34
Martial arts	4
Rugby	6
Soccer	51
Softball	9
Tennis	7
Track and field	12
Ultimate frisbee	21
Volleyball	14
X‐country	8
Missing	237

### 2.2. Measures

#### 2.2.1. GAD‐7 and PHQ‐9

GAD‐7 is a 7‐item scale with each item scored from 0 (*not at all*) to 3 (*nearly every day*), allowing for a total score ranging from 0 to 21 to measure anxiety severity [18]. PHQ‐9 consists of 9 items using the same scoring range, resulting in scores from 0 to 27 to measure depression severity [19]. Each of these scales has standardized norms that can be used to classify the degree of severity for anxiety [18] and depression [19].

#### 2.2.2. ImPACT

Twenty‐two symptoms were used from the ImPACT. From these symptoms, four different clusters were derived: affective (e.g., emotional responses such as sadness or irritability), sleep (e.g., disruptions in sleep patterns), cognitive cluster (e.g., challenges with concentration and memory), and physical (e.g., symptoms like headaches and dizziness). The use of these clusters is consistent with previous research that supports the construct validity of affective symptoms relative to depression and the stability of cluster structures, providing psychometric support for screening applications [10, 11, 20].

### 2.3. Procedure

This is a cross‐sectional research design. Baseline assessments were conducted as part of standardized preseason screenings in preparation for the upcoming season. This included using the ImPACT, GAD‐7, and PHQ‐9 scales. Participants underwent a battery of other health assessments; however, other evaluations and nonmental health‐related tests were excluded. Assessments were conducted in a computer lab with 2–10 participants simultaneously to minimize interactions and maximize focus.

### 2.4. Data Analysis

Four hierarchical regression models were applied to assess the predictive capability of the ImPACT symptom clusters on anxiety and depression outcomes (total score and those scoring above moderate levels), controlling for baseline mental health scores and demographic variables such as gender and ADHD status. The dependent variables were the PHQ‐9 total score for depression and the GAD‐7 total score for anxiety. Predictor variables were the ImPACT symptom cluster scores, along with sex and ADHD status as covariates. The models aimed to parse out the unique contributions of each symptom cluster to the variance in mental health scores. Statistical analyses were conducted using SPSS software, with a significance level set at *p* < 0.05. The regression analysis involved stepwise inclusion of variables: first, demographic controls, followed by baseline mental health scores, and finally, the symptom scores from the ImPACT test.

## 3. Results

### 3.1. Depression and Anxiety Severity

Depression severity indicated that 87.9% (*n* = 492) of participants exhibited none to minimal depressive symptoms, while 8.9% (*n* = 50) reported mild depression. Moderate depression was observed in 3.0% (*n* = 17) of participants, and only one participant (0.2%) fell within the moderately severe range. No participants scored in the severe depression category.

Similarly, anxiety severity revealed that 87.5% (*n* = 490) of participants reported none to minimal anxiety symptoms, 8.9% (*n* = 50) had mild anxiety, 2.7% (*n* = 15) had moderate anxiety, and 0.9% (*n* = 5) exhibited severe anxiety. The correlation between anxiety and depression scores was *r* = 0.739, *p* < 0.001. Forty‐two of the participants (7.5%) reported symptoms for both anxiety and depression at or above the mild level, and 54 were at or above the mild level for either anxiety or depression.

Comparatively, estimates among collegiate student‐athletes report similar prevalence to our sample. Using the Center for Epidemiologic Studies‐Depression (CES‐D), another study reported clinically elevated depressive symptoms in 23.7% of Division I athletes (moderate–severe: 6.3%) in a large single‐institution sample, with a higher risk in women [4]. More recently, a multi‐institution study found 22.3% of NCAA Division I/II athletes at risk for depression by CES‐D and 12.5% above norms for anxiety by the State‐Trait Anxiety Inventory [21]. Relative to the broader, non‐athlete, college population, recent national surveys, also employing the GAD‐7 and PHQ‐9, indicate higher overall prevalence with 41% screening positive for depression (PHQ‐9 moderate to severe) and 36% screening positive for anxiety (GAD‐7, moderate to severe) on the PHQ‐9 [22].

#### 3.1.1. Influence of ADHD and Sex on Depression, Anxiety, and Concussion Symptom Clusters

Two separate multivariate analysis of variance (MANOVA) were conducted. The first examined PHQ‐9 depression totals and GAD‐7 totals as dependent variables with sex and ADHD status as factors, while the second examined ImPACT symptom cluster scores as dependent variables with the same factors. See Table 3 for means and standard deviations. For the MANOVA for depression and anxiety, a significant main effect was found for both gender (Pillai’s Trace = 0.024, *F*(2, 555) = 6.776, *p* = 0.001, partial *η* ^2^ = 0.024) and ADHD status (Pillai’s Trace = 0.016, *F*(2, 555) = 4.423, *p* = 0.012, partial *η* ^2^=0.016), but not a significant interaction (Pillai’s Trace = 0.002, *F*(2, 555) = 0.602, *p* = 0.548, partial *η* ^2^ = 0.002). A significant effect for gender was observed, with females showing higher scores on both depression (*F* (1, 556) = 8.717, *p* = 0.003, partial *η* ^2^=0.015) and anxiety (*F* (1, 556) = 13.420, *p* < 0.001, partial *η* ^2^ = 0.024). For ADHD status, those with ADHD had higher scores on depression (*F*(1, 556) = 8.596, *p* = 0.004, partial *η* ^2^=0.015), but not anxiety (*F* (1, 556) = 3.228, *p* = 0.073, partial *η* ^2^ = 0.006).

**TABLE 3:** Differences in depression and concussion symptom clusters by sex and ADHD status.

	Depression	Anxiety	Cognitive	Physical	Affective	Sleep
Total group	1.7 ± 2.7	1.7 ± 3.0	1.0 ± 2.1	0.6 ± 1.4	1.4 ± 2.7	1.7 ± 2.4
*Sex*						
Female (*n* = 276)	2.0 ± 2.9	2.3 ± 3.4	1.0 ± 2.2	0.6 ± 1.3	1.8 ± 3.0	1.9 ± 2.5
Male (*n* = 284)	1.4 ± 2.4	1.2 ± 2.4	0.9 ± 1.9	0.6 ± 1.5	1.1 ± 2.3	1.5 ± 2.2

*Diagnosed ADHD*						
No (*n* = 473)	1.6 ± 2.5	1.7 ± 2.9	0.8 ± 1.9	0.5 ± 1.2	1.4 ± 2.6	1.6 ± 2.3
Yes (*n* = 87)	2.4 ± 3.4	2.1 ± 3.4	2.0 ± 2.7	1.0 ± 2.2	1.7 ± 3.0	1.9 ± 2.6

A second MANOVA was conducted to examine the effects of gender and ADHD status on concussion symptom clusters (cognitive, physical, affective, and sleep‐related symptoms). Similarly, a significant main effect for gender (Pillai’s Trace = 0.018, *F*(4, 553) = 2.553, *p* = 0.038, partial *η* ^2^ = 0.018) and ADHD status was found (Pillai’s Trace = 0.056, *F*(4, 553) = 8.170, *p* < 0.001, partial *η* ^2^ = 0.056), but not a significant interaction between gender and ADHD status (Pillai’s Trace = 0.006, *F*(4, 553) = 0.776, *p* = 0.541, partial *η* ^2^ = 0.006). Subsequent univariate analyses found that the only significant effect for gender was affective cluster (*F* (1, 556) = 6.981, *p* = 0.008, partial *η* ^2^ = 0.12), but not for any other concussion symptom cluster. ADHD status had significant influences on cognitive symptoms (*F* (1, 556) = 27.014, *p* < 0.001, partial *η* ^2^ = 0.046) and physical symptoms (*F* (1, 556) = 7.366, *p* = 0.007, partial *η* ^2^=0.013), but not affective and sleep clusters.

#### 3.1.2. Prediction of Depression Based on Concussion Symptom Clusters

A hierarchical regression model predicting PHQ‐9 total score revealed that ADHD and sex accounted for 2.7% of the variance in depressive symptoms (*R* ^2^ = 0.027, *p* < 0.001; *F* (2, 557) = 7.735, *p* < 0.001). The inclusion of affective symptom clusters significantly increased the explained variance (*R* ^2^ change = 0.226, *p* < 0.001; *F*(1, 556) = 168.144, *p* < 0.001), followed by sleep‐related symptoms (*R* ^2^ change = 0.041, *p* < 0.001; *F*(1, 555) = 32.257, *p* < 0.001) and cognitive symptoms (*R* ^2^ change = 0.018, *p* < 0.001; *F* (1, 554) = 14.858, *p* < 0.001), leading to a final model accounting for 30.6% of the variance (*R* ^2^ = 0.306). See Table 4 for more descriptives of the model.

**TABLE 4:** Predictors of depression with concussion symptom clusters.

	Predictors included	*R* ^2^	Adjusted *R* ^2^	*R* ^2^_change_	*F*_change_	*p*
Step 1	ADHD, sex	0.027	0.024		7.74	< 0.001
Step 2	Affect cluster	0.253	0.249	0.226	168.14	< 0.001
Step 3	Sleep cluster	0.294	0.289	0.041	32.257	< 0.001
Step 4	Cognitive cluster	0.306	0.305	0.018	14.858	< 0.001

A separate analysis predicting PHQ‐9 scores at or above the moderate threshold found no significant predictors.

#### 3.1.3. Prediction of Anxiety Based on Concussion Symptom Clusters

For anxiety, a hierarchical regression model prediction of the GAD‐7 total score showed that ADHD and sex accounted for 3.5% of the variance (*R* ^2^ = 0.035, *p* < 0.001; *F* (2, 557) = 9.985, *p* < 0.001). The affective symptom cluster was the strongest predictor, significantly increasing the explained variance (*R* ^2^ change = 0.257, *p* < 0.001; F (1, 556), *p* < 0.001) leading to a final model accounting for 28.8% of the variance (*R* ^2^ = 0.288). See Table 5 for more descriptives of the model.

**TABLE 5:** Predictors of anxiety with concussion symptom clusters.

	Predictors included	*R* ^2^	Adjusted *R* ^2^	*R* ^2^_change_	*F*_change_	*p*
Step 1	ADHD, sex	0.035	0.031		9.99	< 0.001
Step 2	Affect cluster	0.292	0.288	0.257	201.967	< 0.001

*Mild or higher anxiety*						
Step 1	ADHD, sex	0.021	−0.008		0.732	0.485
Step 2	Affect cluster	0.096	0.055	0.074	5.43	0.023

A separate analysis predicting GAD‐7 scores at or above the moderate threshold found that ADHD and sex alone were not significant predictors (*F* (2, 67) = 0.732, *p* = 0.485), but affective symptoms contributed significantly to the model (*R* ^2^ change = 0.074; *F* (1, 66) = 5.429, *p* = 0.023).

## 4. Discussion

### 4.1. Interpretation and Clinical Meaning

The present study found that ImPACT symptom clusters, especially the affective cluster, are informative for identifying athletes who are at a greater risk of reporting anxiety or depression. While the explained variance is modest, it does support that routine concussion screenings can flag mental health risk. In practice, these clusters can function within baseline workflow as a pragmatic flag that prompts further evaluation. ImPACT may therefore be used, not as a comprehensive evaluation or diagnostic tool to replace validated tests or infer impairment, but as a practical screening aid to identify at‐risk individuals who can follow up with gold standard measures.

When affective or sleep cluster scores are elevated at baseline, administering staff may use the PHQ‐9 and/or GAD‐7 and guide student‐athletes to institutional referral pathways. This two‐part approach provides a feasible course of action to identify at‐risk individuals and may help surface concerns among athletes who are more prone to normalize fatigue, mood changes, or stress in competitive settings. It is therefore important to acknowledge that with quantitative follow‐up measures such as the PHQ‐9 and GAD‐7, athletes may endorse mild or moderate symptoms while continuing to perform well in classes and training. In practice elevated screens should guide staff to hold supportive conversations, including questions about functioning such as sleep regularity and affect. Such an approach balances early identification with the reality of many athletes mastering life demands, even while symptoms are present.

### 4.2. Demographic Influences on Mental Health Outcomes

Demographic factors, particularly sex and ADHD status, significantly influenced mental health outcomes among collegiate athletes. Females exhibited slightly higher mean scores in both depression and anxiety clusters, aligning with earlier research indicating that female athletes report more intense psychological distress [4]. This heightened vulnerability may stem from both biological factors (e.g., hormonal fluctuations) and sociocultural influences (e.g., expectations regarding emotional expression). Meanwhile, participants with a diagnosis of ADHD recorded elevated cognitive and physical symptom scores, suggesting that inattention, impulsivity, and possible executive function deficits may compound their risk for mental health challenges [15, 16].

While these findings are exploratory and should be interpreted as attention points for administrators rather than determinants of care, they may highlight the need for tailored mental health assessments that account for individual factors. Female athletes and those with ADHD may experience overlapping or additive stressors that can either mask or amplify anxiety and depression symptoms. Incorporating demographic variables into early screening practices may thus facilitate more precise identification of at‐risk athletes.

### 4.3. Prevalence of Depression and Anxiety

A substantial proportion of participants reported experiencing depressive or anxious symptoms. Specifically, nearly 9% endorsed mild depressive symptoms, 3% reported moderate levels, and a small fraction indicated moderately severe or higher depression. Likewise, roughly 9% reported mild anxiety, 3% reported moderate anxiety, and under 1% reached severe anxiety thresholds. When focusing on those with above moderate symptom severity (e.g., PHQ‐9 scores ≥ 10 or GAD‐7 scores ≥ 10), the proportion decreased, but these athletes exhibited markedly higher affective and sleep cluster scores. This contrast between mild and more severe cases underscores the importance of identifying not only subclinical symptoms but also the subset of athletes who may require immediate intervention.

By examining how athletes at or above moderate thresholds in depression and anxiety scored on the ImPACT symptom clusters, we were able to better characterize the multifaceted nature of their distress. Those with more pronounced depressive or anxious symptoms displayed higher affective (e.g., sadness, irritability) and sleep (e.g., insomnia, fatigue) cluster scores, indicating the potential value of these clusters as early indicators for athletes who may be nearing clinically significant levels of distress.

### 4.4. Regression Analyses and Predictive Factors

Hierarchical regression models demonstrated that demographic variables (sex and ADHD status) explained a small but significant portion of the variance in both depression and anxiety. Importantly, the addition of the affective symptom cluster substantially increased the explained variance, suggesting that emotions such as irritability, nervousness, or sadness serve as key predictors of mental health outcomes. The sleep cluster also contributed uniquely to the prediction of depression, aligning with existing literature that links disrupted sleep patterns to heightened depressive symptoms [11].

By contrast, the cognitive and physical clusters exhibited somewhat smaller—but still significant—effects in the regression models. This finding points to a complex interplay among different symptom domains, whereby concentration difficulties and somatic complaints may exacerbate or coincide with affective and sleep disturbances. Athletes who accumulate high scores across multiple clusters may be at the greatest risk for more severe forms of depression or anxiety, warranting proactive screening and timely referral to mental health services. Beyond the symptom clusters, there are other variables that probably need to be taken into consideration to identify those who might be struggling with mental health issues. Some of these variables could include fitness status, whether injured, how they are adjusting to college (these measurements are often taken when first transitioning to school), and relationships.

### 4.5. Implications for Screening and Intervention

Taken together, these results support the use of ImPACT symptom clusters for identifying athletes who are at heightened risk for depression and anxiety. Given the additional influence of demographic variables, integrating a brief demographic screening (e.g., inquiring about ADHD status or targeted questions for female athletes) with ImPACT cluster scores could enable more precise identification of athletes requiring intervention. ImPACT is already embedded ubiquitously in near‐universal baseline workflows, which reduces both the stigma and logistical burden of administering mental health screens; leveraging its cluster scores could flag and trigger the use of referrals to tailored gold‐standard screeners. Such an approach holds promise for reducing barriers to care and improving early detection of distress. These screenings often happen early in the transition to the school phase and could be helpful in early identification before athletes begin to build relationships with teammates, coaches, and other athletic staff who might be helpful in providing interventions or support for mental health issues.

### 4.6. Ethics and Transparency

Repurposing a concussion tool for mental‐health screening requires administrators to clearly communicate during onboarding that the baseline assessment may also inform mental‐health risk. Programs should specify what is reviewed, how the privacy of participant information is protected, who sees it, and what happens should the results suggest follow‐up. Consent materials and staff training should emphasize that elevated scores do not intrinsically affect playing status.

### 4.7. Implementation

In practice, when affective or sleep cluster scores appear elevated, administering the PHQ‐9 and GAD‐7 at the same session provides validated measures to inform the most appropriate support route. Athletes with a PHQ‐9 score of at least 10 or a GAD‐7 of at least 10 should be referred to a mental healthcare professional. Any endorsement of PHQ‐9, item 9, should be immediately recognized as a safety concern requiring urgent evaluation according to institutional policy.

### 4.8. Limitations and Future Directions

Although these findings offer valuable insights, several limitations warrant consideration. The cross‐sectional nature of the data limits any causal inferences regarding the direction of relationships between symptom clusters and mental health outcomes. Some concerns also come from the few participants who reported anxiety and depression symptoms, but these percentages were consistent with previous research on this population. Additionally, no validated measures of functional impairment or performance outcomes were collected, limiting the determination of how symptom levels relate to academic or athletic functioning within the cohort. Because academic and sport demands fluctuate across a year, future implementation of the screening workflow could include a preseason baseline, mid‐term, and end‐of‐term to help address symptom oscillations and finding validity. Additional research could explore longitudinal designs to capture changes in symptom clusters over time or investigate additional moderating factors (e.g., sport type, injury history, or social support). Expanding this approach to diverse athlete populations across multiple institutions would also help clarify the generalizability of these findings and assist in specifying a diagnostic cut score for ImPACT to further improve its reliability.

## 5. Conclusion

By emphasizing demographic influences and the predictive utility of ImPACT symptom clusters, the current study offers a framework for more nuanced mental health screening in collegiate athletics. Athletes reporting moderate or higher levels of depression or anxiety demonstrated particularly elevated scores in the affective and sleep domains, hinting at the value of these clusters as early warning signs. In conjunction with ADHD and sex variables, integrating ImPACT‐based assessments into routine protocols may foster timely intervention, reduce underreporting, and ultimately contribute to better mental health outcomes in this high‐risk population.

## Funding

No funding was received for this manuscript.

## Conflicts of Interest

The authors declare no conflicts of interest.

## Data Availability

The data that support the findings of this study are available on request from the corresponding author. The data are not publicly available due to privacy or ethical restrictions.
